# Giant barocaloric effect in hexagonal Ni_2_In-type Mn-Co-Ge-In compounds around room temperature

**DOI:** 10.1038/srep18027

**Published:** 2015-12-17

**Authors:** Rong-Rong Wu, Li-Fu Bao, Feng-Xia Hu, Hui Wu, Qing-Zhen Huang, Jing Wang, Xiao-Li Dong, Guan-Nan Li, Ji-Rong Sun, Fei-Ran Shen, Tong-Yun Zhao, Xin-Qi Zheng, Li-Chen Wang, Yao Liu, Wen-Liang Zuo, Ying-Ying Zhao, Ming Zhang, Xian-Cheng Wang, Chang-Qing Jin, Guang-Hui Rao, Xiu-Feng Han, Bao-Gen Shen

**Affiliations:** 1 Beijing National Laboratory for Condensed Matter Physics & State Key Laboratory of Magnetism, Institute of Physics Chinese Academy of Sciences, Beijing 100190, P. R. China; 2 NIST Center for Neutron Research, National Institute of Standards and Technology, Gaithersburg, Maryland 20899, USA; 3 Department of Materials Science and Engineering, University of Maryland, College Park, MD 20742-2115, USA; 4 Department of Information Materials Science and Engineering, Guilin University of Electronic Technology, Guilin, Guangxi 541004, P. R. China

## Abstract

The most widespread cooling techniques based on gas compression/expansion encounter environmental problems. Thus, tremendous effort has been dedicated to develop alternative cooling technique and search for solid state materials that show large caloric effects. An application of pressure to a material can cause a change in temperature, which is called the barocaloric effect. Here we report the giant barocaloric effect in a hexagonal Ni_2_In-type MnCoGe_0.99_In_0.01_ compound involving magnetostructural transformation, *T*_*mstr*,_ which is accompanied with a big difference in the internal energy due to a great negative lattice expansion(*ΔV/V* ~ 3.9%). High resolution neutron diffraction experiments reveal that the hydrostatic pressure can push the *T*_*mstr*_ to a lower temperature at a rate of 7.7 K/kbar, resulting in a giant barocaloric effect. The entropy change under a moderate pressure of 3 kbar reaches 52 Jkg^−1^K^−1^, which exceeds that of most materials, including the reported giant magnetocaloric effect driven by 5 T magnetic field that is available only by superconducting magnets.

Caloric effects of materials driven by different external fields such as pressure, magnetic field, and electric field are known as barocaloric, magnetocaloric, and electrocaloric effect, respectively. Any change of lattice, spin, electric polarization ordering is accompanied by entropy change, thus the caloric effect can be measured by isothermal entropy change or adiabatic temperature change. It is easily understandable that barocaloric effect is universal noting that an application of pressure on any material can cause a change in lattice ordering and lead to a caloric effect. Actually, the conventional cooling techniques in our daily life or industry applications are based on compression and expansion cycles of gases, but these popular techniques directly or indirectly cause many environmental problems. In the past two decades, the discovery of solid state materials with giant magnetocaloric/electrocaloric/barocaloric effect has indeed promoted the development of solid state refrigeration techniques^1^,^2^,^3^,^4^,^5^,^6^. Generally, barocaloric effect is small for most of solid state materials^7^,^8^, such as Pr_*x*_La_1−*x*_NiO_3_, Ce_3_Pd_20_Ge_6_, EuNi_2_(Si_*x*_Ge_1−*x*_)_2_, CeSb, and HoAs, because the applied pressure cannot produce substantial changes in the structure and/or magnetic ordering. The entropy change produced by a moderate pressure is not enough to fulfill the requirement of the practical refrigeration. Here, we report a sizable barocaloric effect in a Mn-Co-Ge-In compound, which originates from a pressure-driven orthorhombic-hexagonal magnetostructural transition. To the best of our knowledge, this is the first time that the giant barocaloric effect has been observed in a system with a hexagonal Ni_2_In-type structure. High resolution neutron diffraction experiments reveal that the phase transition is accompanied with a significant re-construction of crystal structure, noting the lattice change can be as large as *ΔV/V* ~ 3.9%, which exceeds that of the most other caloric materials with a lattice contribution. Such a significant re-construction of crystal structure brings about a big difference in the internal energy. Careful refinements on the structure reveal that the interlayer Mn-Mn distance behaves critically sensitive to pressure, indicating the origin of the pressure-driven magnetostructural transition and giant barocaloric effect.

Currently, magnetic refrigeration is being considered to be a competitive solid state refrigeration technique around room temperature due to the discovery of giant magnetocaloric materials, such as Gd_5_(Si,Ge)_4_ ref. 9, MnFeP_1−*x*_As_*x*_ ref. 10, MnAs ref. 11, La(Fe,Si)_13_ ref. 12,13 and NiMn-based Heusler alloys^14^,^15^,^16^. A common feature of these materials is the concurrent change of crystallographic and magnetic properties during phase transitions. In other words, magnetic phase transition always takes place along with a discontinuous change in lattice parameters and/or crystal symmetry. For most of them, both magnetic field and pressure can drive the first-order phase transition. Thus these materials should also display a barocaloric effect, as predicted by some theoretical investigations^17^. Manosa *et al.*^4^,^5^ observed a considerable barocaloric effect near room temperature in La-Fe-Co-Si and Ni-Mn-In systems, in which the entropy changes (*ΔS*) are 8.6 Jkg^−1^K^−1^ under 2.1 kbar and 24.4 Jkg^−1^K^−1^ under 2.6 kbar, respectively, reaching 75% and 90% of the total (11.4 Jkg^−1^K^−1^ and 27.0 Jkg^−1^K^−1^). Such *ΔS* magnitude has exceeded the elastic heating effect of most materials and is also larger than the reported magnetocaloric effect (MCE) induced by magnetic fields that are available with permanent magnets^4^. Recently, Matsunami *et al.* reported giant barocaloric effect enhanced by the frustration in the antiferromagnetic Mn_3_GaN. The entropy change reaches 22.3 Jkg^−1^K^−1^ under a hydrostatic pressure change of 1.39 kbar ref. 6.

Ternary compounds MM’X with hexagonal Ni_2_In-type structure have recently attracted much attention due to their magnetic shape memory effect and possible large magnetocaloric effect related to the magnetostructural coupling^18–24^. As a member of MM’X family, the stoichiometric MnCoGe alloy does not show magnetostructural coupling. It undergoes a diffusionless martensitic structural transition, *T*_*stru*_, from a Ni_2_In-type hexagonal structure (space group *P6*_*3*_*/mmc*) to a TiNiSi-type orthorhombic structure (space group *Pnma*) at *T*_stru_ ~ 420 K and a separated ferromagnetic ordering transition at a lower temperature *T*_*c*_ ~ 345 K^18^. Fortunately, both magnetic interaction and crystallographic stability are sensitive to chemical pressures, such as substitution, doping, or interstitials. Introducing atoms with different radii and valence electrons can simultaneously tune magnetic and crystallographic transitions and make the two separated transitions to overlap with each other. As a result, magnetostructural coupling can be created. For the MM’X family, the austenitic hexagonal phase has a smaller unit cell volume than the martensitic orthorhombic phase. This fact indicates that introducing smaller atoms or vacancies may probably stabilize hexagonal phases and shift *T*_*stru*_ to a lower temperature. Indeed, magnetostructural transition has been experimentally realized through introducing atom vacancies or smaller atoms, such as MnCo_1−x_Ge ref. 20, Mn_1−x_CoGe ref. 21, Mn_1−x_Cr_x_CoGe ref. 22. However, the change of local environments is not the sole route to affect *T*_*stru*_. Valence electron concentration (*e/a*) may also play an important role. We found that introducing larger atoms with fewer valence electrons can also lower *T*_*stru*_ and create the magnetostructural transition. Indium (In) atom (2.00 Å, 5 s^2^5p^1^) has a larger atomic radius but fewer valence electrons than Mn(1.79 Å, 3d^5^4 s^2^), Co(1.67 Å, 3d^7^4 s^2^), or Ge(1.52 Å, 4 s^2^4p^2^). We found that the replacement of Mn, Co, or Ge by a little amount of In can create magnetostructural coupling. Particularly, the magnetostructural transition temperature shows a monotonous decrease with increasing In doping for MnCoGe_1−x_In_x_ (Fig. S1, Supplementary material S1). Here we chose MnCoGe_0.99_In_0.01_ compound that owns a transition temperature near room temperature to study the barocaloric effect.

## Results

From the temperature dependence of magnetization (M-T curve) measured under a low field of 0.01 T upon heating and cooling for MnCoGe_0.99_In_0.01_ (Fig. S1), we found that the magnetic transition between ferromagnetic (FM) and paramagnetic (PM) phases occurs at *T*_*C*_ ~ 308 K. The thermal hysteresis, ~8 K, indicates the first-order nature of the transition involving magnetostructural coupling (*T*_*stru*_ = *T*_c_, denoted as *T*_*mstr*_ thereafter). Here, the transition temperature is defined as the point where the largest slope occurs upon heating. Generally, hysteresis behavior is related to many intrinsic and extrinsic factors (Supplementary material S1). Thermal activation model was usually considered to investigate dynamic behaviors^25^,^26^. A narrow hysteresis gap indicates the energy barrier, which closely correlates with the electronic band structure and the nucleation factors, is smaller than that of other materials with a large hysteresis gap. In view of applications, a small hysteresis gap is beneficial for a practical use.

**Figure 1:**
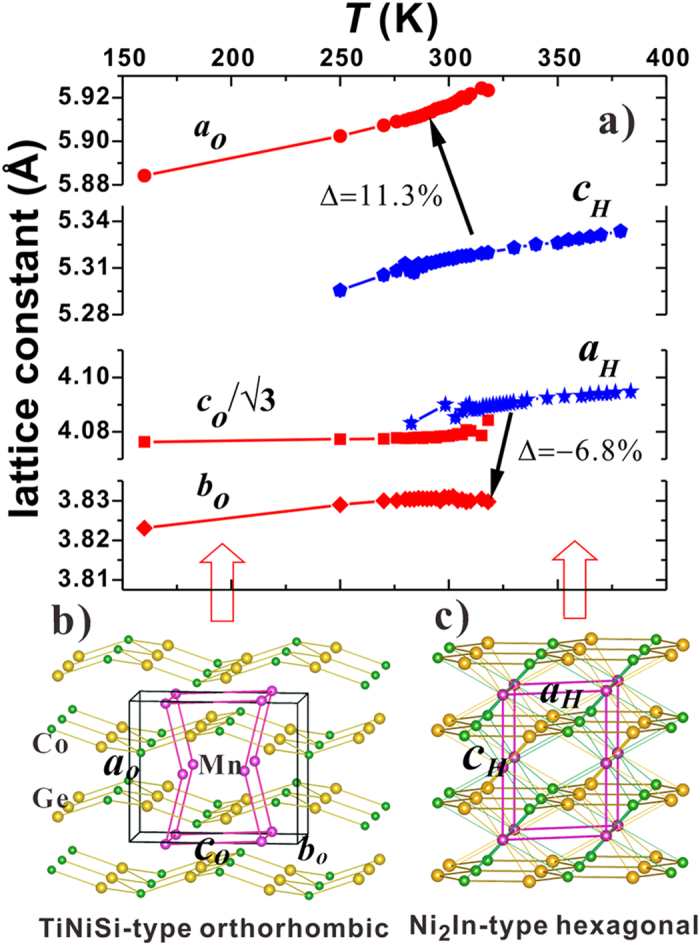
Lattice constants and unit-cell volume for MnCoGe_0.99_In_0.01_, and the sketches of the orthorhombic and hexagonal structures. (**a**) Variation of lattice constants and unit-cell volume of the hexagonal and orthorhombic structure with temperature, and the sketches of (**b**) orthorhombic and (**c**) hexagonal structures. The black lines in (**b**) enclose one unit cell of TiNiSi-type orthorhombic structure while the purple lines in (**c**) enclose one unit cell of Ni_2_In-type hexagonal structure. Upon phase transformation from hexagonal to orthorhombic phase, the unit cell of hexagonal structure transforms into the zigzag-type structure in the orthorhombic phase, as indicated by purple lines in (**b**).

To confirm and examine the details of the concurrent magnetic and structural transitions, we performed neutron powder diffraction (NPD) studies on the crystal and magnetic structures as the functions of temperature, external magnetic field, and pressure. It is noticeable that, from the NPD results, with the disappearance of magnetic ordering the sample undergoes a structural transformation from the orthorhombic martensite to the hexagonal austenite (Fig. S2a,b, Supplementary material S2). Meanwhile, an abrupt drop of unit cell volume, *ΔV/V* = (*V*_ortho_/2 − *V*_hex_)/*V*_hex_ ≈ 3.9%, occurs (Note: the unit-cell of the two phases has the relationship *V*_ortho_ = 2*V*_hex_ ref. 27). This result evidences that a transition occurs between FM orthorhombic and PM hexagonal structure. Figure 1a displays the refined lattice constants of the hexagonal and orthorhombic structure with temperature. The hexagonal structure expands pronouncedly along the c-axis (*c*_*H*_) by ~11.3% while contracts significantly along the a-axis (*a*_*H*_) by ~−6.8% for MnCoGe_0.99_In_0.01_, noting that the lattice constants of the hexagonal and orthorhombic structure have relations as *a*_*o*_ = *c*_*H*_, *b*_*o*_ = *a*_*H*_, *c*_*o*_ = *√3a*_*H*_^24^. This fact indicates that the crystal structure undergoes a significant re-construction during the martensitic phase transformation. To visualize the structural transformation and intuitively understand the refined results, the sketches of TiNiSi-type orthorhombic and Ni_2_In-type hexagonal structures are displayed in Fig. 1b,c, respectively. The representative NPD pattern collected at 304 K in the phase transformation region is presented in Supplementary materials (Fig. S2-c), including the difference plot. Lattice parameters and phase ratio can be derived from refinements.

We note that the change of unit cell volume, *ΔV/V* ~ 3.9%, across the phase transformation for MnCoGe_0.99_In_0.01_ is much larger than that of most other magnetocaloric materials with lattice contributions, such as MnAs (|*ΔV/V*| ~ 2.2%)^28^, LaFe_11.2_Co_0.7_Si_1.1_(|*ΔV/V*| ~ 1.3%)^29^, FeRh(|*ΔV/V*| ~ 0.9%)^30^, Gd_5_Si_1.8_Ge_2.2_(|*ΔV/V*| ~ 0.4%)^31^, Mn_3_GaN(|*ΔV/V*| ~ 1.0%)^6^. Such a large difference in the unit cell volume implies a big difference in the internal energy, thus a large latent heat should occur during the transition.

Another interesting feature is that the temperature region where two phases coexist reaches ~80 K around the *T*_*mstr*_ (from ~250 K to ~330 K) (Fig. S2a, Supplementary material S2), indicating the structural transformation of the sample proceeds in a wide temperature range. This behavior will make the caloric effect occur in a wide temperature range, which is favorable for practical applications.

**Figure 2:**
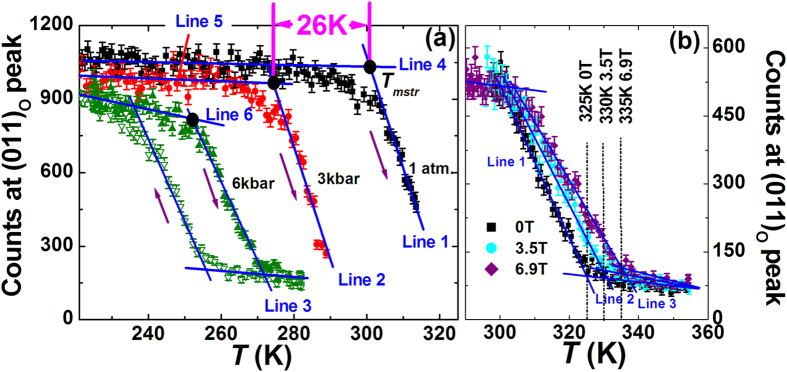
Counts at (011)_O_ reflections under hydrostatic pressure and magnetic field for MnCoGe_0.99_In_0.01_. (**a**) Neutron intensity variations (with error bars) of (011)_O_ peak in the orthorhombic phase as a function of temperature under different pressures. The black plot is the warming path under ambient pressure. The red plot is the warming path under 3 kbar. The olive plots are the warming and cooling paths under 6 kbar, respectively. (**b**) Neutron intensity variations (with error bars) of the (011)_O_ reflection of the orthorhombic phase as a function of temperature under different magnetic fields. The black plot was obtained in the absence of a magnetic field. The cyan and purple plots were obtained under a field of 3.5 T and 6.9 T, respectively.

To study the effect of different external stimuli on the magnetostructural transformation, we monitored the peak intensity of the (011)_O_ reflection in the orthorhombic phase as a function of temperature under different hydrostatic pressures (Fig. 2a) and magnetic fields (Fig. 2b). Consistent with the results shown in Fig. S2a, the counts at (011)_O_ peak drop gradually with increasing temperature, signifying the structural transformation from the orthorhombic martensitic to the hexagonal austenitic phase across over a wide temperature range. External pressure stabilizes the hexagonal phase through shortening the Mn-Mn interlayer distance and strengthening the covalent bonding between Mn-Mn atoms. A hydrostatic pressure of 6 kbar can make a significant decrease of *T*_*mstr*_ (Here, the value of *T*_*mstr*_ is defined as the solid circle point shown in Fig. 2a) from 300 K down to 254 K, and the driving rate is as high as 7.7 K/kbar. On the contrary, the external magnetic field produces opposite effect. It stabilizes the FM orthorhombic phase and pushes the *T*_*mstr*_ to higher temperatures at a moderate rate of 1.4 K/T. Thus, the corresponding barocaloric effect should be of inverse while magnetocaloric effect is conventional, similar to the case in LaFe_11.33_Co_0.47_Si_1.2_ ref. 4, *i.e.*, the sample absorbs heat upon an application of pressure whereas releases heat under an applied magnetic field.

**Figure 3:**
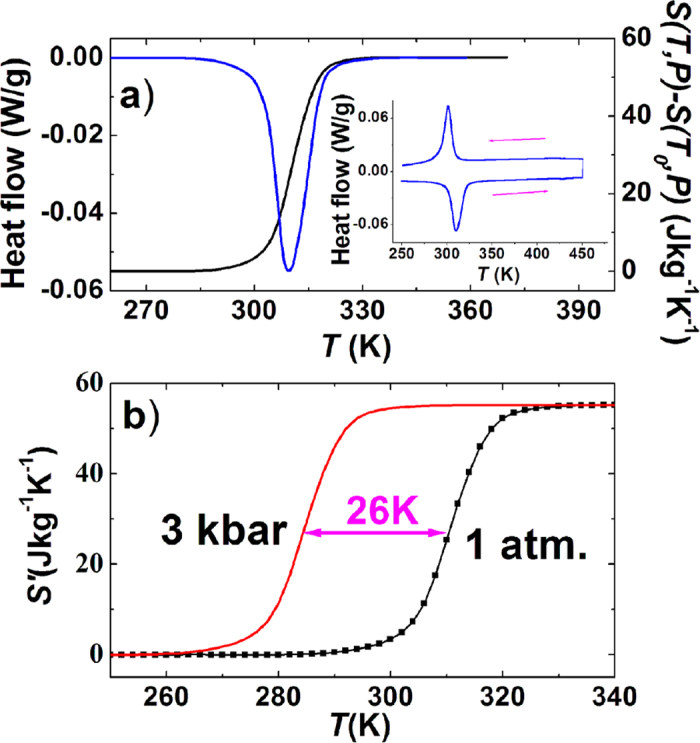
Heat flow and entropy for MnCoGe_0.99_In_0.01_. (**a**) Temperature dependence of heat flow (blue, endothermic curve) and entropy (black) Effect of epitaxial strain on small-polaron hopping conduction in Pr_0.7_(Ca_0.6_Sr_0.4_)_0.3_MnO_3_ thin filmsAppl. Phys. Lett. 106, 102406(2015) AIP publishing Mar. 2015 (42), *S*′*(T,P)* (referenced to the value at 260 K) measured under ambient pressure without a magnetic field. The inset shows the DSC curves on heating and cooling, where arrows indicate the cooling/warming paths. (**b**) Temperature dependence of the entropy, *S*′*(T,P)*, with neglecting the contributions of *Cp*. The entropy measured under ambient and 3 kbar pressure were plotted in black and red, respectively.

To evaluate the heat effect under external stimuli, we performed the calorimetric measurements using a differential scanning calorimeter (DSC), which has been considered to be a reliable and best way to evaluate caloric effect for a system with a first-order phase transition^4^,^5^,^32^,^33^ (Supplementary material S3). The inset to Fig. 3a presents the heat flow as a function of temperature measured under ambient pressure without magnetic field. From these data, the entropy change across the phase transition can be calculated using the following equation,


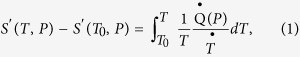


where 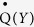 is the heat flow, 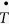 is the ramping rate of temperature, and *P* is the hydrostatic pressure. The integral is computed after properly subtracting the baseline, which means the contribution from the specific heat capacity *C*_*P*_ is completely neglected and only the phase transition process is focused^4^,^5^. The temperature dependent entropy (*S*′*(T,P)-T* curve) with neglecting *C*_*P*_, along with the DSC endothermic curve, is shown in Fig. 3a. The total entropy change was found to be as large as 55 Jkg^−1^K^−1^ across transition. This value exceeds those of most magnetocaloric materials evaluated by the same way, such as twice larger than that of Ni-Mn-In(27 Jkg^−1^K^−1^)^5^, 5 times larger than that of LaFe_11.33_Co_0.47_Si_1.2_ (11.4 Jkg^−1^K^−1^)^4^, and more than twice larger than that of Mn_3_GaN(22.3 Jkg^−1^K^−1^)^6^.

However, the absolute value of total entropy as a function of temperature cannot be obtained solely by DSC technique although it can accurately measure the latent heat and entropy change across the first-order transition^4^,^5^. We performed *Cp* measurements from 2 K to 360 K by PPMS calorimeter (Fig. S3a, Supplementary material S4). By plus both contributions from *Cp-T* measured by PPMS (neglecting the *Cp* caused by latent heat due to its inaccuracy in phase transition region^33^) and the heat flow measured by DSC (neglecting *Cp* in the non-phase transition region), the total entropy (*S-T*) curves can be obtained.

**Figure 4:**
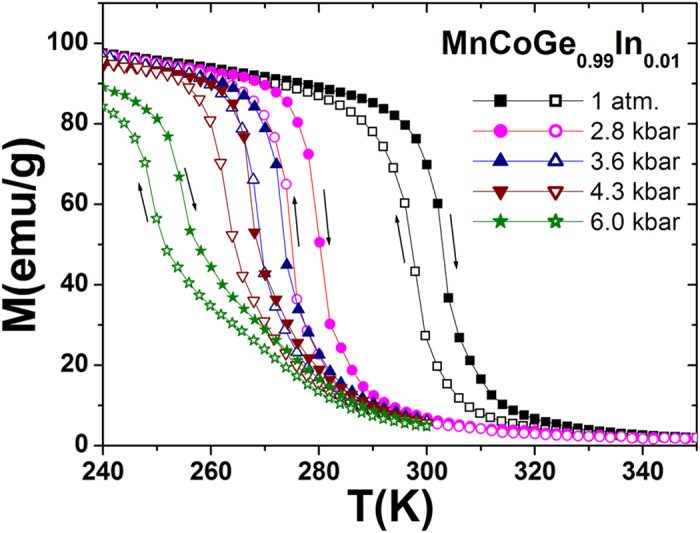
Magnetic measurements under pressure for MnCoGe_0.99_In_0.01_. Temperature dependent magnetization (M-T curve) on warming and cooling at different hydrostatic pressures (1 atm., 2.8 kbar, 3.6 kbar, 4.3 kbar, 6.0 kbar) under 0.5 T magnetic field. Arrows indicate the warming and cooling paths.

From Fig. 2a, it is noticeable that a 3 kbar pressure shifts the *T*_*mstr*_ from 300 K down to 274 K, and more importantly it does not impact the transition width, the slope, and the dependence of lattice on temperature in the non-phase transition region noting the slope of the fitting lines on I_(011)o_ vs. temperature keeps nearly the same as those under the ambient pressure, *i.e.*, line 2 parallels line 1, line 5 parallels line 4. The difference of the unit cell volume between the orthorhombic and hexagonal phases under 3 kbar (Δ*V*/*V = *(*V*_ortho_/2 − *V*_hex_)/*V*_hex_ ~ 3.95%) maintains the same as the value under an ambient pressure (~3.9%). Continuously increasing pressure to 6 kbar makes the *T*_*mstr*_ further decrease, but the transition becomes slow and the dependence of the lattice on temperature behaves differently in the non-transition region (line 3 is not parallel to lines 2 and 1, and line 6 is not parallel to lines 5 and 4, Fig. 2a). Moreover, we measured the temperature dependent magnetization (M-T curve) at different hydrostatic pressures (1 atm., 2.8 kbar, 3.6 kbar, 4.3 kbar, 6.0 kbar) under 0.5 T magnetic field, as shown in Fig. 4. It is found that the thermal hysteresis gap remains nearly unchanged and the *M-T* curves in the phase transition region under *P* ≤ 3.6 kbar is nearly parallel to the case under 1 atm., but the *M-T* curve becomes notably slow as the applied pressure reaches 6.0 kbar. This behavior is completely consistent with the variations of Bragg peak observed in Fig. 2a. Caron *et al.*^22^ also demonstrated a similar result in a MnCoGe-based compound Mn_0.93_Cr_0.07_CoGe. They found that an applied pressure lower than 3.7 kbar can retain the transition width and slope but a 5 kbar pressure slows down the transition.

**Figure 5:**
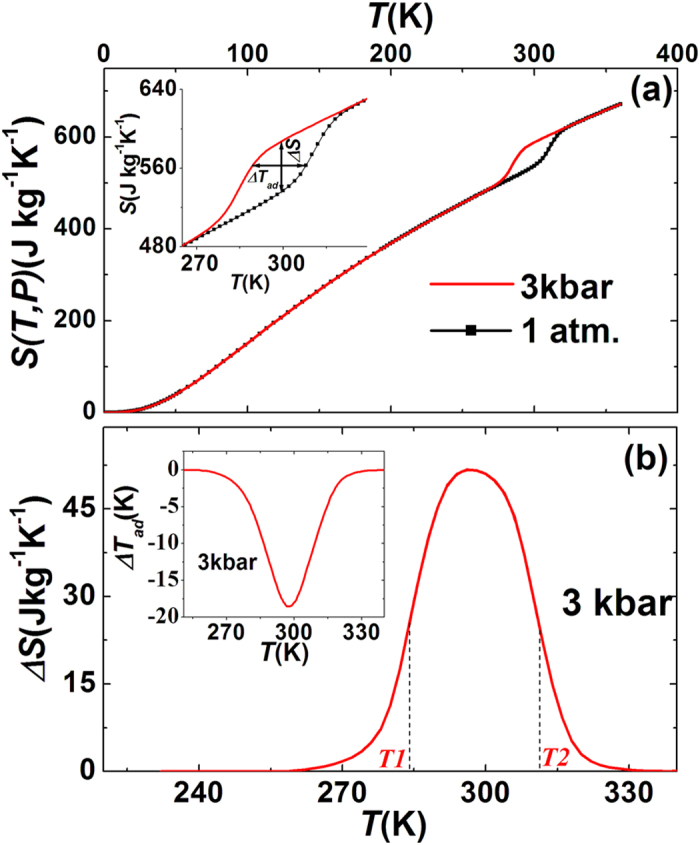
Barocaloric effects of MnCoGe_0.99_In_0.01_. (**a**) Temperature dependence of the total entropy, *S(T,P)*, under different pressures. The entropy measured under ambient and 3 kbar pressure were plotted in black and red, respectively. Inset shows the details of total entropy. (**b**) Temperature dependence of entropy in a pressure change of 0–3 kbar, where the *T*_*1*_ and *T*_*2*_ represent the temperature at which the entropy change is half of the peak. The inset shows the adiabatic temperature change as a function of temperature under 3 kbar.

For present MnCoGe_0.99_In_0.01_ under 3 kbar, the unchanged lattice and magnetization indicates that the lattice elastic energy and magnetic exchange energy would not be affected by a 3 kbar pressure in the non-phase transition regions. In this situation, it should be safe to obtain the *S*′*(T,P)-T* curve under 3 kbar with neglecting *C*_*P*_ through simply shifting the *S*′*(T,P)-T* curve at the ambient pressure down to a lower temperature by 26 K (calculated from the driving effect of 3 kbar pressure on *T*_*mstr*_, 300 K−274 K = 26 K), as shown in Fig. 3b. Moreover, it should also be reasonable to assume that the basic *Cp*-*T* under 3 kbar with neglecting the latent heat should maintain the same as that under the ambient pressure. Thus the total *S-T* curves under the ambient pressure and 3 kbar can be safely obtained by adding the *S*′*-T* relationships and the contributions from the basic *Cp*-*T*, as the black and red curves shown in Fig. 5a, respectively (Supplementary material S4).

For other systems such as NiMnIn and LaFeCoSi with similar magnetostructural or magnetoelastic transitions, the obtained *S*′*(T,P)-T* curves derived from DSC with neglecting *C*_*P*_ were also found to be in parallel to each other even a 2.6 kbar pressure was applied^4^,^5^. The applied pressure of 2.6 kbar/2.1 kbar shifts the *T*_*mstr*_ of Ni-Mn-In/LaFe_11.33_Co_0.47_Si_1.2_ to a higher/lower temperature by 4.5 K/14 K. The specific driving effect for different materials should be mainly related to the differences in the lattice instability and the strength of the lattice spin interactions under different external fields. Meanwhile, the transformation kinetics should also play a contributory role. For the present MnCoGeIn system, the transition becomes slow as the pressure reaches 6 kbar (Figs 2a and 4). In this case, it is inappropriate to obtain the *S(T,P)-T* relationships using the above simple method.

From these total entropy-temperature (*S-T*) curves shown in Fig. 5a (inset shows the details), the entropy change *ΔS* and the adiabatic temperature change *ΔT*_*ad*_ can be safely deduced^34^, and is shown in Fig. 5b and its inset, respectively. It can be seen that the entropy change is indeed inverse and the maximal *ΔS* is about 52 Jkg^−1^K^−1^ (299 K), and the maximal *ΔT*_*ad*_ attains to be ~18.5 K under an application of 3 kbar pressure. This pressure-induced *ΔS* value already reaches 94% of the maximal value corresponding to the total entropy change of 55 Jkg^−1^K^−1^ for the transition. To verify the obtained *ΔS* is reliable, we also evaluated it by using Clausius-Clapeyron equation 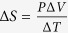
(Supplementary material S5), the commonly accepted method for the first-order transition systems^35^, where *ΔV* is the change of unit cell volume across transition, and *ΔT* is the shift of transition temperature triggered by pressure. The calculated *ΔS* is 56.6 J/kgK under 3 kbar based on the available *ΔV* and *ΔT* from the neutron diffraction results shown in Fig. S2a and Fig. 2a. This value agrees well with that of the total entropy change of 55 Jkg^−1^K^−1^ (Fig. 3b). Such a good consistency undoubtedly shows that the entropy change evaluated based on DSC measurements is completely reliable. However, for a practical first-order system, the phase transition usually occurs in a finite temperature width, which smoothes the *S-T* curves. Thus the evaluated *ΔS* (~52.0 ± 5.6 Jkg^−1^K^−1^ under 3 kbar, i.e. ~17.3 ± 1.9 Jkg^−1^K^−1^kbar^−1^, details about the error evaluation can be found in supplementary material S5) based on DSC does not reach 100% of the total entropy change. Such a *ΔS* is larger than those of most materials driven by either pressure or magnetic field, such as Gd_5_Si_2_Ge_2_ (18 Jkg^−1^K^−1^, 276 K, 0–5 T; i.e. 3.6 Jkg^−1^K^−1^T^−1^; 13 Jkg^−1^K^−1^ under 3 kbar, i.e. 4.3 Jkg^−1^K^−1^kbar^−1^)^9^,^36^, Fe_49_Ph_51_(12 Jkg^−1^K^−1^T^−1^; 12 Jkg^−1^K^−1^kbar^−1^)^37^, MnFeP_0.45_As_0.55_ (18 Jkg^−1^K^−1^, 308K, 0–5 T; i.e. 3.6 Jkg^−1^K^−1^T^−1^)^10^, LaFe(_0.88_Si_0.12_)_13_H_y_(23 Jkg^−1^K^−1^, 195 ~ 336 K, 0–5 T; i.e. 4.6 Jkg^−1^K^−1^T^−1^)^13^, MnAs(30 Jkg^−1^K^−1^, 318 K, 0–5 T; i.e. 6.0 Jkg^−1^K^−1^T^−1^)^11^, Ni_50_Mn_37_Sn_13_(18 Jkg^−1^K^−1^, 300 K, 0–5 T; i.e. 4.6 Jkg^−1^K^−1^T^−1^)^15^, Ni-Mn-In(24.4 Jkg^−1^K^−1^, 293 K, 0–2.6 kbar; i.e. 9.4 Jkg^−1^K^−1^kbar^−1^)^5^, LaFe_11.33_Co_0.47_Si_1.2_(8.6 Jkg^−1^K^−1^, 230 K, 0–2.1 kbar; i.e. 4.1 Jkg^−1^K^−1^kbar^−1^)^4^, and Mn_3_GaN (22.3 Jkg^−1^K^−1^, 283 K, 0–1.39 kbar; i.e. 16.0 Jkg^−1^K^−1^kbar^−1^)^6^. For the *ΔS* in the unit per kbar, MnCoGe_0.99_In_0.01_ shows comparable *ΔS* to that of Mn_3_GaN. For the well-known magnetocaloric materials, the required magnetic field to produce a giant magnetocaloric effect is usually larger than 3 T, which can be available only by superconducting magnets, however a moderate pressure up to 3 kbar can be easily accessed by the present approach.

Furthermore, we directly measured the pressure-induced adiabatic temperature change (*ΔT*_*ad*_) at room temperature *RT* (note: the local *RT* was ~298 K when the measurements were performed, which is nearly the same as the peak temperature ~299 K of the entropy change, see Fig. 5b). It was observed that the sample indeed cooled down upon pressure, indicating the barocaloric effect is of inverse. The detected maximal *ΔT*_*ad*_ was about 9.4 K under 3 kbar (Fig. S4, Supplementary material S6). This value is considerably large though it is only half of the maximal *ΔT*_*ad*_ (~18.5 K) evaluated from the *S-T* curves. This mismatch is understandable considering the unavoidable leakage heat due to the contact solid pressure medium, which will certainly lead to an underestimation of *ΔT*_*ad*_ (details can be found in the following section of Methods). The pressure-induced *ΔT*_*ad*_ value is also attractive compared to other systems, *i.e.* ~4.5 K for Ni-Mn-In (2.6 kbar pressure)^5^, ~4–10 K for Fe_49_Ph_51_ (2.5 kbar pressure)^38^, ~6.2 K for Ni-Mn-In-Co (2 T magnetic field)^16^, ~2.2 K for LaFe_11.33_Co_0.47_Si_1.2_ (2 kbar pressure)^4^, ~15 K for Gd_5_Si_2_Ge_2_ (5 T magnetic field)^9^, and ~12 K for PbZr_0.95_Ti_0.05_O_3_ (480 kV cm^−1^ electric field)^2^.

For the effect of magnetic field on MnCoGe_0.99_In_0.01_, an application of magnetic field shifts the *T*_*mstru*_ to a higher temperature, indicating the magnetocaloric effect is conventional (the material will release heat to environments upon applying magnetic field, opposite to the effect of pressure). However, we found that a 3.5 T magnetic field has already made the structural transformation slow due to spin-lattice coupling, indicated by the gradual decrease in the slope of the fitting lines (lines 1, 2, 3) with increasing magnetic field (Fig. 2b). For such situation it is dangerous to evaluate entropy change by simply shifting the *S*′*(T,P)-T* curve (with neglecting *C*_*P*_). However, considering the low driving rate of magnetic field on the *T*_*mstr*_ (1.4 K/T), the conventional magnetocaloric effect even under 6.9 T should be significantly smaller than the total transition entropy change.

## Discussion

The hexagonal Ni_2_In-type MnCoGe_0.99_In_0.01_ compound undergoes a magnetostructural transformation between the ferromagnetic orthorhombic and paramagnetic hexagonal structure. The big difference in the internal energy contributed from the change of thermal energy, lattice elastic energy and magnetic exchange energy across the phase transition should be responsible for the giant barocaloric effect, noting the high sensitivity of the magnetostructural transition to pressure. It is known that the phonon frequencies, lattice elastic and magnetic exchange energy can be expressed in terms of Debye temperature, elastic modulus and magnetic coupling coefficient, respectively. High resolution neutron diffraction experiments reveal that the phase transition is accompanied with a negative lattice expansion as large as *ΔV/V* ~ 3.9%, which exceeds that of the most other giant caloric materials with lattice contribution. Such a significant re-construction of crystal structure will definitely change the phonon frequencies (thermal energy), lattice elastic energy, as well as the magnetic exchange coupling energy through altering the atomic position/symmetry, bond length, and the width of the effective 3d bands, thus produce a big difference in the internal energy across the phase transformation.

Previous investigations on MnNiGe-based compounds with the same hexagonal structure revealed^19^ that the interlayer distance and the strengthening of covalent bonding between Mn-Mn and Ni-Ge atoms played key roles in stabilizing the hexagonal austenitic structure based on the valence-electron localization function (ELF) calculations. In parallel, we examined the Mn-Mn and Co-Ge distances with respect to the temperature and pressure for the present compound. Our careful refinements based on the high resolution neutron diffraction revealed that the bond-length of all the nearest and the second nearest *d1* and *d2* for Mn-Mn/Co-Ge neighbors linearly contracts upon cooling to the temperatures near and above the *T*_*mstr*_ (Fig. S5-1, S5-2, Supplementary material S7). The contractive rate is about −1.12 * 10^−4^ Å/K and −3.80 * 10^−5^ Å/K for *d1*(Mn-Mn) and *d1*(Co-Ge), respectively. However, the situation is quite different while applying hydrostatic pressure although pressure can also make the lattice contract. We surprisingly find that the *d1* and *d2* have different variations for both Mn-Mn and Co-Ge neighbors with respect to hydrostatic pressure measured at a constant temperature of 259 K (Fig. S5-3, Supplementary material S7). The *d1*(Mn-Mn) contracts at a rate of −2.9 × 10^−2^ Å/kbar, which is about 2 orders of magnitude faster than the contraction caused by temperature(−1.12 × 10^−4^ Å/K), while *d1*(Co-Ge) remains nearly unchanged. The rapidly shortening of the Mn-Mn interlayer distance (*d1*) with pressure will strengthen the covalent bonding between Mn-Mn atoms, which should be largely responsible for the stabilization of the hexagonal austenitic structure, and also affect the width of the effective 3d bands that contributes to the magnetic coupling. Here, it is noteworthy that the sample is polycrystalline and the hydrostatic pressure is applied isotropically using helium gas as a medium. This fact indicates that the Mn-Mn interlayer distance (*d1*) is more sensitive to pressure, which can rapidly reduce *d1*(Mn-Mn) and thus lead to a significant shift of the *T*_*mstr*_to a lower temperature by 7.7 K/kbar. The high sensitivity of the magnetostructural transition to pressure and the big difference in the internal energy across phase transformation lead to the giant barocaloric effect.

In comparison to pressure, magnetic field is not an effective way to trigger the magnetostructural transition noting the small driving rate of magnetic field on the *T*_*mstr*_ (1.4 K/T). This fact indicates that the phase transition can be driven more easily by the pressure-induced crystallographic change than by the magnetic field-induced spin-lattice coupling. This is a common feature particularly for the MnCoGe-based alloys^22^. In these systems, the driving force of the magnetostructural transition is the crystallographic transition and the magnetic transition occurs cooperatively. Generally, the cooperative-type transition involves two ordering parameters, one acts as a dominant role while another changes in a cooperative way although the transition occurs simultaneously for both ordering parameters^39^. The magnetocrystalline transitions are usually the cooperative type. For most magnetocaloric materials undergoing magnetostructural or magnetoelastic transformations, such as GdSiGe ref. 40, La(Fe,Co,Si)_13_ ref. 4, and Ni-Mn-In ref. 5, both the crystallographic change induced by hydrostatic/chemical pressure and the magnetic field can drive the transitions. However, it is hard to distinguish which one acts as the dominant driving force and which one acts cooperatively. As a result, these materials simultaneously show the giant magnetocaloric and barocaloric effects. However, for the system whose magnetostructural/magnetoelastic transition is only sensitive to the pressure but not to the magnetic field, giant barocaloric effect determined by the latent heat should definitely occur regardless of the magnetocaloric effect.

Moreover, for the present MnCoGe_0.99_In_0.01_, a pressure less than 3 kbar can only shift the transition starting point but not affect the transition path, while a higher pressure of 6 kbar slows down the transition. The applied magnetic field affects the transition path more significantly than the applied pressure. We try to understand these phenomena by considering the lattice instability and the transformation kinetics under different external fields. As discussed above, thermal activation model was usually considered to investigate dynamic behaviors for the materials with first-order transitions^25^,^26^. The energy barrier in the model closely correlates with the electronic band structure and nucleation factors. For the present system, the applied pressure largely shortens the Mn-Mn interlayer distance (*d1*) (Fig. S5-3), and thus stabilizes the hexagonal austenitic structure and pushes the transition to lower temperature. The change of atomic local environments definitely affects the width of the effective 3d bands that contributes to the magnetic coupling and lattice-spin interaction. As the applied pressure is over a critical value, strong lattice deformation and structural instability appear, thus the thermal activation energy and the critical nucleation size and nucleation process, which indicate the coexistence of two phases^25^,^26^, will be largely affected. As a result, the coexistent region of two phases may be extended and structural transformation occurs in an extended temperature range under a high pressure. However, the applied magnetic field shows opposite effect. It stabilizes the orthorhombic martensitic structure and drives the transition to higher temperature. The change of local environments should be largely different from the case under pressure, thus the activation energy and the critical nucleation size during phase transition should be also different. Although we cannot present a quantitative explanation for the different transition paths under the pressure and magnetic field, the different transformation kinetics relative to electronic band structures^26^ and the different lattice deformations/instabilities caused by the pressure and magnetic field should play a dominant role.

In summary, giant barocaloric effect is successfully realized in a hexagonal Ni_2_In-type MnCoGeIn alloy by utilizing a large negative expansion of lattice(*ΔV/V* ~ 3.9%) across a magnetostructural transformation, which is accompanied by a significant difference in the internal energy and thus a large latent heat. The observed barocaloric effect is of inverse and the material cools down upon applying hydrostatic pressure, similar to the case in LaFeCoSi. The caloric effect under a moderate pressure of 3 kbar in a MnCoGeIn alloy exceeds those of the most reported materials driven by a similar pressure or a 5 T magnetic field. These results demonstrate that pressure can be used to maximize the contribution of lattice to the caloric effect for a material with a structural/magnetostructural transition that exhibits a high sensitivity to pressure rather than the magnetic field. By maximizing and optimizing the lattice contribution, we can enhance the solid caloric effect effectively and readily. We anticipate that the present study can inspire further interest in exploring novel solid state refrigeration materials and promote the development of solid state refrigeration technology around the ambient temperature.

## Methods

### Sample preparation and magnetic measurements

MnCoGeIn alloys were prepared by repeatedly arc-melting appropriate amounts of starting materials in high-purity argon atmosphere (99.996%) with a base pressure of 10^−4^ Pa. The commercial purities of Mn, Co, Ge, In are 99.9 wt%, 99.9 wt%, 99.999 wt%, and 99.99 wt%, respectively. The obtained ingots were wrapped separately with Mo foil and subsequently homogenized in a sealed quartz tube under vacuum of 10^−4^ Pa at 875 K for 6 days, then cooled down to room temperature in the furnace. Magnetic measurements were performed using a superconducting quantum interference device magnetometer (SQUID-VSM). Magnetic measurements under hydrostatic pressure were performed using a commercial pressure cell, which works at pressures up to 10 kbar and mineral oil is used as the pressure transmitting medium.

### Neutron diffraction measurements

High-resolution powder-diffraction data were collected using the BT1 32-detector neutron powder diffractometer at the NIST Center for Neutron Research (NCNR). A Cu (311) monochromator was used to produce the monochromatic neutron beam with the wavelength of λ = 1.5403(2) Å. For the structure refinement, data were collected in the 2-theta range of 3–168° with a step size of 0.05°. Collimations before and after the monochromator and after the sample were 60′, 20′, and 7′, respectively. Closed Cycle Refrigerator (CCR) was used in temperature dependent measurements ranging from 100 K to 400 K. For the magnetic field experiments, a superconducting magnet was employed to produce a vertical magnetic field up to 7 T, and the measurements were performed in the temperature range from 200 K to 360 K controlled by a top loading CCR. The pressure measurements were carried out using Al (7075-T6) high pressure sample cell and the pressure was applied up to 6.15 kbar with helium gas as the pressure medium. The Rietveld refinements were performed using GSAS program^41^.

### Caloric measurements

Heat flow was carried out using a commercial differential scanning calorimeter (DSC, Q200, TA instruments. Such identification fosters understanding but does not imply recommendation or endorsement by the NIST and CAS). Before each measurement, the calorimeter was carefully calibrated using standard data of sapphire. The ramping rate of 3 K/min was taken for both heating and cooling processes. Specific heat capacity *C_P_* was measured using PPMS calorimeter (Quantum Design) [Supplementary material S4].

To carry out measurements of the adiabatic temperature change *ΔT*_*ad*_ under pressure, a home-made device that can work only at room temperature was set up since no commercial device is available at the moment. Fortunately, the peak temperature (299 K) of the giant barocaloric effect locates exactly at the room temperature (Fig. 5b), thus we can examine the peak Δ*T_ad_* under pressure. The details are as follows: We put about 800 mg MnCoGeIn sample in a 6mm diameter cylindrical BN container surrounded by pyrophyllite, and placed a Pt-1000 resistance thermometer in the centre of the sample, and then measured the temperature change under pressure, which was applied by a six-anvil hydraulic press. Repeated measurements showed that the sample indeed cools down under application of pressure, indicating the barocaloric effect is of inverse. Fig. S4 in Supplementary material S6 displays the measured temperature as a function of time as a 3 kbar pressure is applied (the pressure can be reached within 3s, see the blue arrow in Fig. S4). The detected maximal *ΔT*_*ad*_ under 3 kbar is about 9.4 K, not reaching the maximal *ΔT*_*ad*_ (~18.5 K) evaluated from the *S-T* curves. Repeated measurements revealed similar *ΔT*_*ad*_ values. This result can be understandable considering the bad adiabatic environments noting that the pressure is applied through solid direct contact mode by a six-anvil hydraulic press. Furthermore, due to the relaxation of the heat exchange, the measured temperature gradually decreases (not stepwise change), noting that the Pt resistance thermometer is plastic-coated in order to insulate it from the sample.

## Additional Information

**How to cite this article**: Wu, R.-R. *et al.* Giant barocaloric effect in hexagonal Ni_2_In-type Mn-Co-Ge-In compounds around room temperature. *Sci. Rep.* **5**, 18027; doi: 10.1038/srep18027 (2015).

## Supplementary Material

Supplementary Information
