# Corrigendum: Giant barocaloric effect in hexagonal Ni_2_In-type Mn-Co-Ge-In compounds around room temperature

**DOI:** 10.1038/srep22826

**Published:** 2016-03-29

**Authors:** Rong-Rong Wu, Li-Fu Bao, Feng-Xia Hu, Hui Wu, Qing-Zhen Huang, Jing Wang, Xiao-Li Dong, Guan-Nan Li, Ji-Rong Sun, Fei-Ran Shen, Tong-Yun Zhao, Xin-Qi Zheng, Li-Chen Wang, Yao Liu, Wen-Liang Zuo, Ying-Ying Zhao, Ming Zhang, Xian-Cheng Wang, Chang-Qing Jin, Guang-Hui Rao, Xiu-Feng Han, Bao-Gen Shen

Scientific Reports 5: Article number: 18027;10.1038/srep18027 published online: 12172015; updated: 03292016

This Article contains errors.

In the legend of Figure 3.

"(**a**) Temperature dependence of heat flow (blue, endothermic curve) and entropy (black) Effect of epitaxial strain on small-polaron hopping conduction in Pr_0.7_(Ca_0.6_Sr_0.4_)_0.3_MnO_3_ thin filmsAppl. Phys. Lett. 106, 102406(2015) AIP publishing Mar. 2015 (42), *S*′*(T, P)* (referenced to the value at 260 K) measured under ambient pressure without a magnetic field."

should read:

"(**a**) Temperature dependence of heat flow (blue, endothermic curve) and entropy (black), *S*′*(T, P)* (referenced to the value at 260 K) measured under ambient pressure without a magnetic field."

In the Methods section under subheading ‘Sample preparation and magnetic measurements’,

"The obtained ingots were wrapped separately with Mo foil and subsequently homogenized in a sealed quartz tube under vacuum of 10^−4^ Pa at 875 K for 6 days, then cooled down to room temperature in the furnace."

should read:

"The obtained ingots were wrapped separately with Mo foil and subsequently homogenized in a sealed quartz tube under vacuum of 10^−4^ Pa at 875 °C for 6 days, then cooled down to room temperature in the furnace."

