# Supplementary material for: Giant barocaloric effect in hexagonal Ni2In-type Mn-Co-Ge-In compounds around room temperature
Source: Sci Rep. 2015 Dec 17;5:18027. doi: 10.1038/srep18027 (PMC4682185; doi:10.1038/srep18027)
Supplement: Supplementary Information [file srep18027-s1.doc]

**SUPPLEMENTAL MATERIAL**

Giant barocaloric effect in hexagonal Ni_2_In-type Mn-Co-Ge-In compounds around room temperature

Rong-Rong Wu^1,2^, Li-Fu Bao^1^, Feng-Xia Hu^1^*, Hui Wu^2,3^, Qing-Zhen Huang^2^, Jing Wang^1^*, Xiao-Li Dong^1^, Guan-Nan Li^1^, Ji-Rong Sun^1^, Fei-Ran Shen^1^, Tong-Yun Zhao^1^, Xin-Qi Zheng^1^, Li-Chen Wang^1^, Yao Liu^1^, Wen-Liang Zuo^1^, Ying-Ying Zhao^1^, Ming Zhang^1^, Xian-Cheng Wang^1^, Chang-Qing Jin^1^, Guang-Hui Rao^4^, Xiu-Feng Han^1^, and Bao-Gen Shen^1^

^1^ Beijing National Laboratory for Condensed Matter Physics, Institute of Physics, Chinese Academy of Sciences, Beijing 100190, P. R. China

^2^ NIST Center for Neutron Research, National Institute of Standards and Technology, Gaithersburg, Maryland 20899, USA

^3^ Department of Materials Science and Engineering, University of Maryland, College Park, MD 20742-2115, USA

^4^ Department of Information Materials Science and Engineering, Guilin University of Electronic Technology, Guilin, Guangxi 541004, P. R. China

*Corresponding email: [fxhu@iphy.ac.cn](mailto:fxhu@iphy.ac.cn), [wangjing@iphy.ac.cn](mailto:wangjing@iphy.ac.cn)

**S1. M-T curves of MnCoGe_1-x_In_x_ compounds**

Fig. S1 displays t tructure. changee magntion ofAle of on r 000000000000000000000000000000000000000000000000000000000000000000000000000000000000TTTt emperature dependent magnetization (M-T curves) measured under a low field of 0.01T on heating and cooling for MnCoGe_1-x_In_x_ compounds with different In doping x=0.01, 0.02, 0.03. All samples show thermal hysteresis, signifying the first-order nature of phase transition involving magnetostructural coupling. The magnetostructural transition temperature, *T_mstr_*, decreases monotonously with increasing In doping, which should be resulted from the competition of the changes in the valence electron concentration (e/a) and local environments. In view of the high interest in room temperature refrigeration, we choose MnCoGe_0.99_In_0.01_ compound with *T_mstr_* around room temperature to study the barocaloric effect. The thermal hysteresis around *T_mstr_* is 8K for this composition.


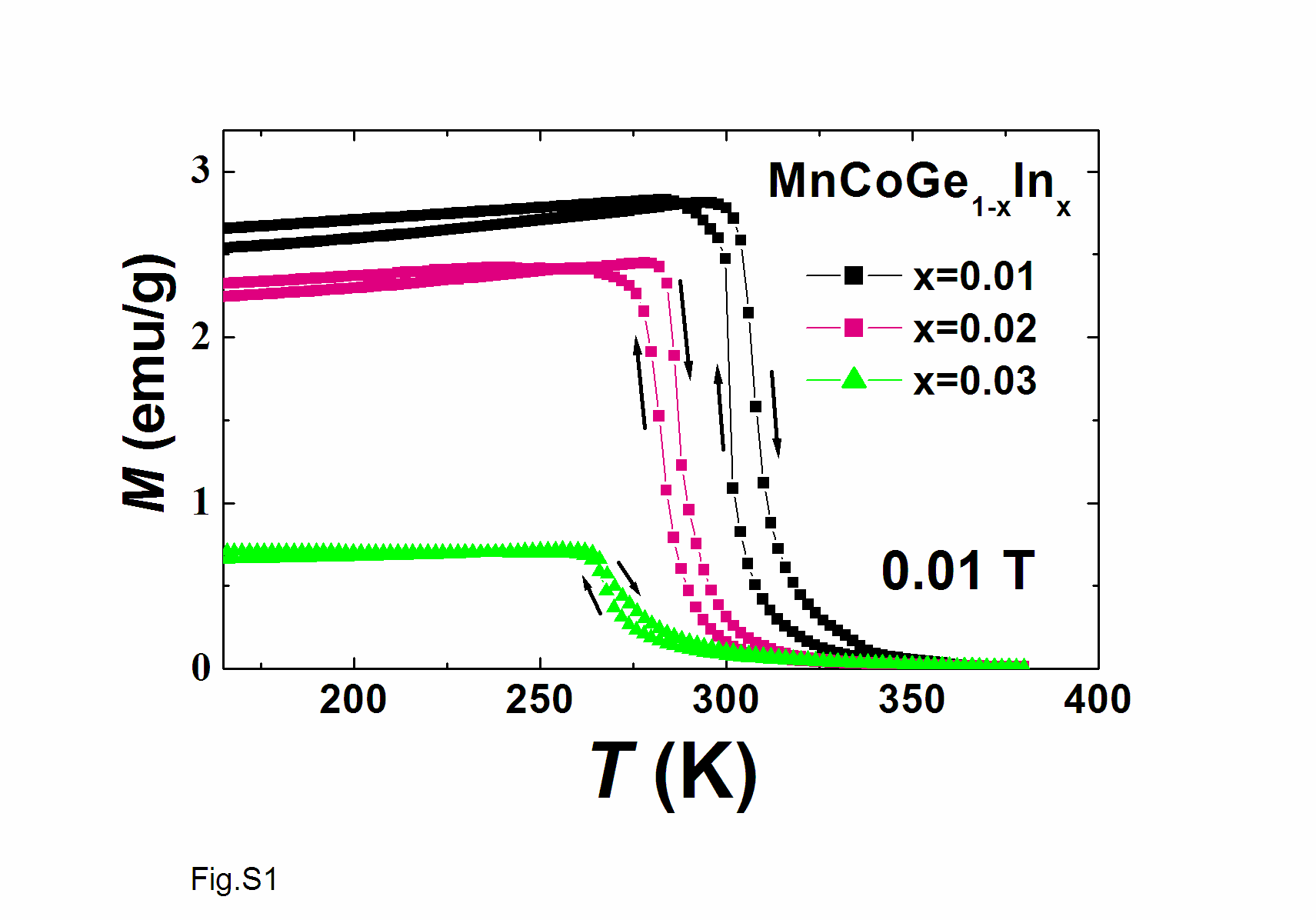


Fig. S1 Magnetization as a function of temperature under a field of 0.01 T for MnCoGe_1-x_In_x_ with different In doping. Arrows indicate the cooling/warming paths.

Generally, hysteresis behavior is related to many intrinsic and extrinsic factors. Intrinsic ones usually include band structure, impurity and nucleation factors, and strain effect. Extrinsic ones mainly refer to the thermal equilibrium and the situation of heat transfer during measurements, which directly relates to the deviation of temperature detector from sample, temperature rate, and etc. During our measurements by using commercial SQUID-VSM, the temperature ramping rate is 2K/s, while the sample mass is ~2 mg. Hence, extrinsic factors involving thermal equilibrium should be negligible, and intrinsic factors should play a key role for the observed hysteresis behaviors. For the magnetocaloric systems with first-order magnetostructural transitions, thermal activation model was usually considered to investigate dynamic behaviors.^25,26^ The energy barrier in the model, which characterizes the hysteresis gap, closely correlates with the electronic band structure and nucleation factors. For example, the magnetic coupling in the Gd_5_Si_2_Ge_2_ system with magnetostructural phase transformation is via itinerant conduction electrons across the (Si,Ge)–(Si,Ge) covalent bonds. As the magnetostructural transformation is triggered by temperature or magnetic field, half of these bonds are broken and reformed in the temperature or magnetic field cycles, thus the energy barrier is closely connected with the magnetic part of the electronic band structure, and that to reforming the (Si,Ge)–(Si,Ge) bonds.^26^ A narrow hysteresis gap indicates the energy barrier, which closely correlates with the electronic band structure and the nucleation factors, is smaller than that of other materials with a large hysteresis gap. In view of applications, a small hysteresis gap is beneficial for a practical use.

**S2. NPD result as a function of temperature**

To confirm and examine the details of the concurrent magnetic and structural transitions, we performed neutron powder diffraction (NPD) studies on the crystal and magnetic structures as functions of temperature, external magnetic field, and pressure. Fig. S2a and b depict the evolution of the unit cell volumes and phase fractions with temperature, together with the magnetic moments of Mn and Co in the orthorhombic phase for MnCoGe_0.99_In_0.01_ with magnetostructural coupling. The refined magnetic moments (Mn: 3.2 μ_B_, Co: 1.0μ_B_) in the orthorhombic phase agree well with the reported values^24^. It is noticeable that with the disappearance of magnetic order the sample undergoes a structural transformation from the orthorhombic martensite (space group: *Pnma*) to the hexagonal austenite (space group: *P6_3_/mmc*). Meanwhile, an abrupt unit cell volume drop of *ΔV/V*=(*V*_ortho_/2-*V*_hex_)/*V*_hex_≈3.9% occurs (Note: the unit-cell of the two phases has the relationship *V*_ortho_ = 2*V*_hex_ [Ref.27]). This fact evidences that a transition occurs between FM orthorhombic and PM hexagonal structure.

Another interesting feature is that the temperature region where martensitic and austenitic phases coexist reaches ~80 K around the *T_mstr_* (from ~250 K to ~330 K) (Fig.S2a), indicating the structural transformation lasts over a wide temperature range instead of at one point. For real materials, the first-order transition usually takes place in a finite temperature region, not at one specific temperature, due to thermal fluctuation or possible inhomogeneity of samples. The wide temperature region of martensitic transformation in ternary MM’X systems has been regarded as a character of t**hermodynamic equilibrium-type martensitic transition in some previous studies.^19^** The wide temperature region of phase transformation will make the caloric effect occur in a wide temperature range, which is favorable for practical applications.


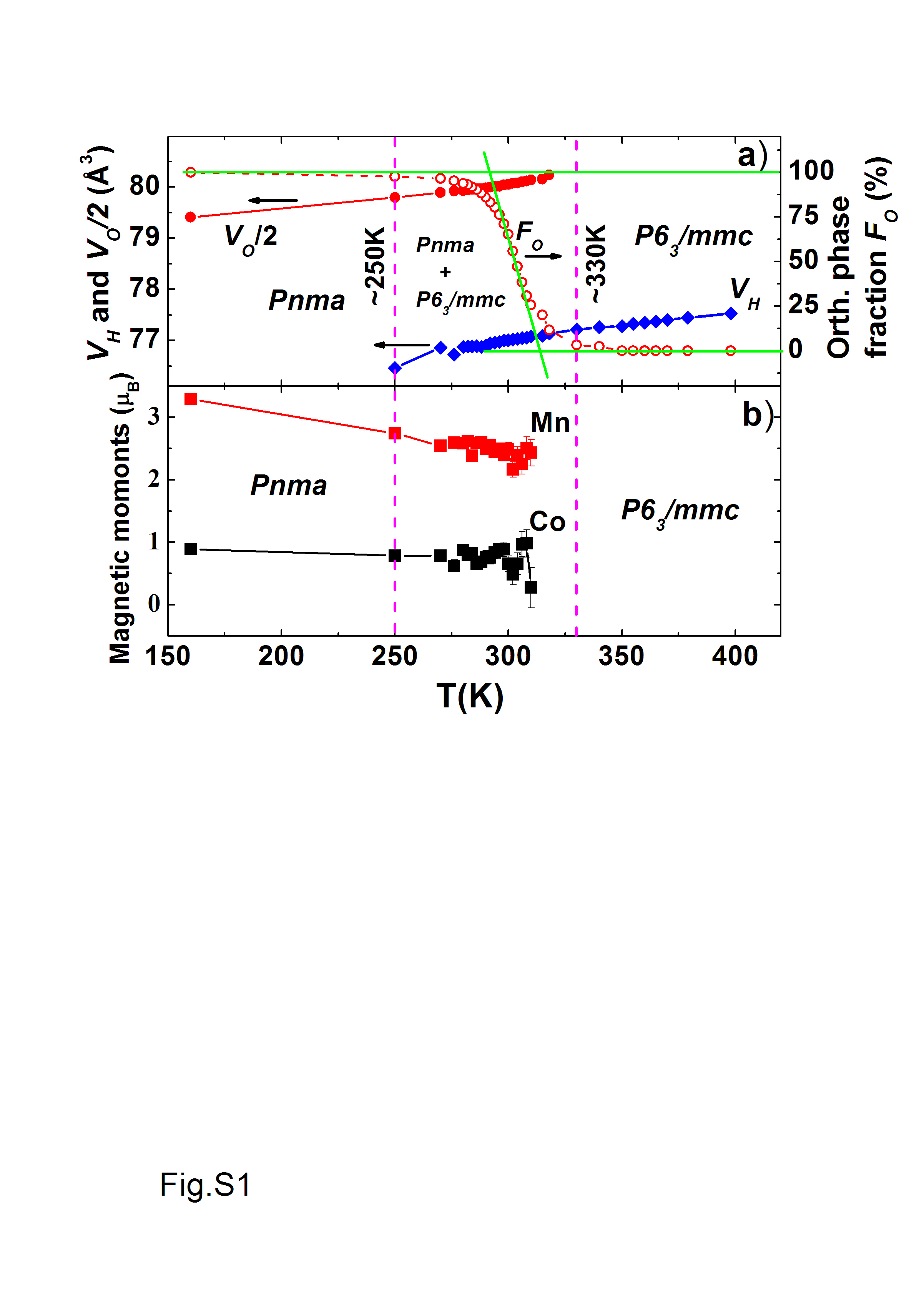


Fig.S2. a)Temperature dependences of the unit cell volume of the hexagonal phase (*V_H_*, blue rhombus), half of unit cell volume of the orthorhombic phase (*V_O_/2*, red solid circle) and orthorhombic phase fraction (*F_O_*, red empty circle), and b) Temperature dependence of the magnetic moments of Mn (red) and Co (black) atoms in the orthorhombic phase for the MnCoGe_0.99_In_0.01_. Since the number of the chemical formula per unit cell is 4 for the orthorhombic phase and 2 for the hexagonal phase, a half of the orthorhombic cell volume *V*_O_/2 is used to compare with the hexagonal unit cell volume *V*_H_ in the plot.

A representative NPD pattern collected at 304 K in the phase transformation region is presented in Fig.S2-c, including the difference plot. Lattice parameters and phase ratio can be derived from refinements. We found that the weight fractions of the orthorhombic and the hexagonal phases are 47.2% and 52.8%, respectively, at 304K.


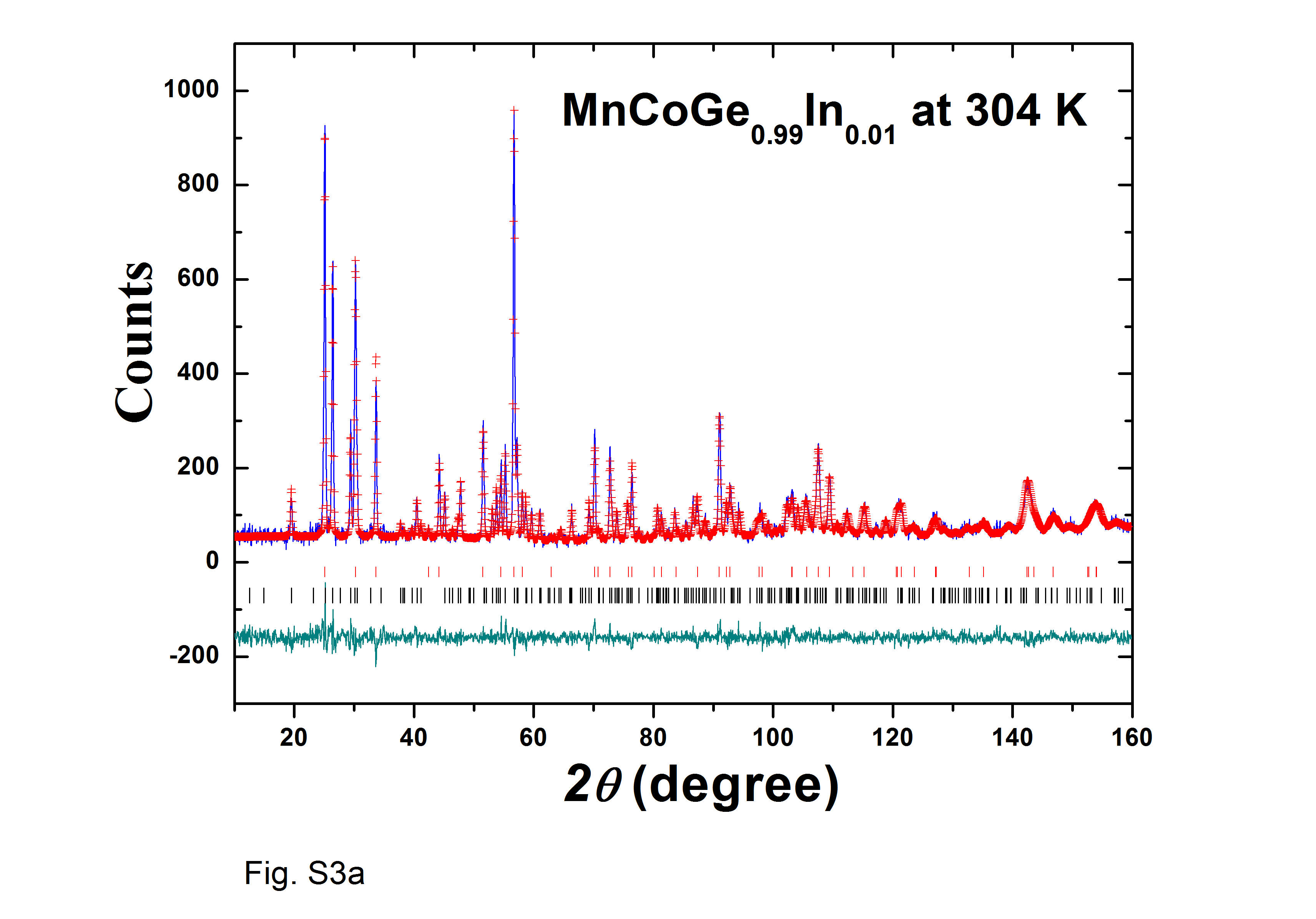


Fig.S2-c Observed (red crosses) and calculated (blue lines) intensities of data collected at 304K where hexagonal and orthorhombic structures coexist. Vertical lines indicate the angular positions of the diffraction for the hexagonal structure (red) and orthorhombic structure (black). Differences are shown in the low part of the plots (dark cyan lines).

**S3. DSC measurements for Caloric effect**

We performed caloric measurements by differential scanning calorimetry (DSC), which has been considered to be a reliable and best way to evaluate caloric effect for a first-order phase transition.^4,5^ Detailed studies carried out by Mañosa et al.^32^ indicated that the best-suited calorimetric technique to measure entropy changes at first-order phase transitions is differential scanning calorimetry (DSC) rather than the devices designed to measure *Cp*. Lashley et al.^33^ examined PPMS calorimeter (Quantum Design), which is popularly used to conduct calorimetric experiments, and found “the system is appropriate to measure *Cp* near second-order transitions accurately, but it is unable to provide accurate measurements of *Cp* near a first-order phase transition. Such an inaccuracy arises from the fact that, due to the latent heat, the temperature-decay curves cannot be described by a single relaxation time constant”. Using a purpose-built DSC calorimeter, caloric effect was successfully evaluated for a number of materials.^4,5^

**S4. Details of *ΔS*, *ΔT_ad_* evaluation**

Fig.S3a displays the *Cp*-*T* (black curve) measured by PPMS from 2K to 360K under ambient pressure. The blue curve represents the *Cp-T*, where *Cp* caused by the latent heat is neglected due to its inaccuracy in phase transition region[ref.33; *Adv. Mater.* ***21****, 3725 (2009)*], from which a basic *S-T* curve ignoring the contribution of the latent heat can be obtained by a numerical integration $S(T,P)=\int_{0}^{T} \frac{C(P,T)}{T}dT$, as shown in Fig.S3b (the red curve).

**From the** high resolution neutron diffraction **(**Fig.2a) and magnetic measurements under pressure (Fig.4), it is noticeable that the 3kbar pressure does not impact transition width and the slope. Moreover, the difference of the unit cell volume between the orthorhombic and hexagonal phases under 3 kbar (*ΔV*/*V =(V*_ortho_/2-*V*_hex_)/*V*_hex_~3.95 %) also maintains nearly the same as the value(~3.9 %) under ambient pressure. The unchanged dependence of lattice and magnetization on temperature indicates that the lattice elastic energy and magnetic exchange energy would not be impacted by a 3kbar pressure in the non-phase transition regions. In this situation, it should be safe to assume that the basic *Cp*-*T* (with neglecting the contributions from the latent heat in the phase transition region) under 3kbar should remain the same as that under the ambient pressure.

Therefore, by combining the *S-T* (blue curve in Fig.S3b obtained from *C_P_*-PPMS, ignoring the contribution of the latent heat) and the *S’-T* curves with neglecting *C_P_* (Fig.3b, obtained from heat flow-DS*C*), the total *S-T* curves under ambient pressure and 3kbar can be obtained, as shown in black and red curves in Fig.S3b, respectively. From these *S-T* curves, the entropy change *ΔS* and the adiabatic temperature change *ΔT_ad_* can be safely deduced ^34^ [upper inset of Fig.S3b], as shown in Fig.5b and its inset, respectively. The maximal *ΔS* is 52 Jkg^-1^K^-1^, reaching 94% of the maximal value corresponding to the total entropy change of 55 Jkg^-1^K^-1^ for the transition, while the maximal *ΔT_ad_* under 3 kbar is 18.5K.


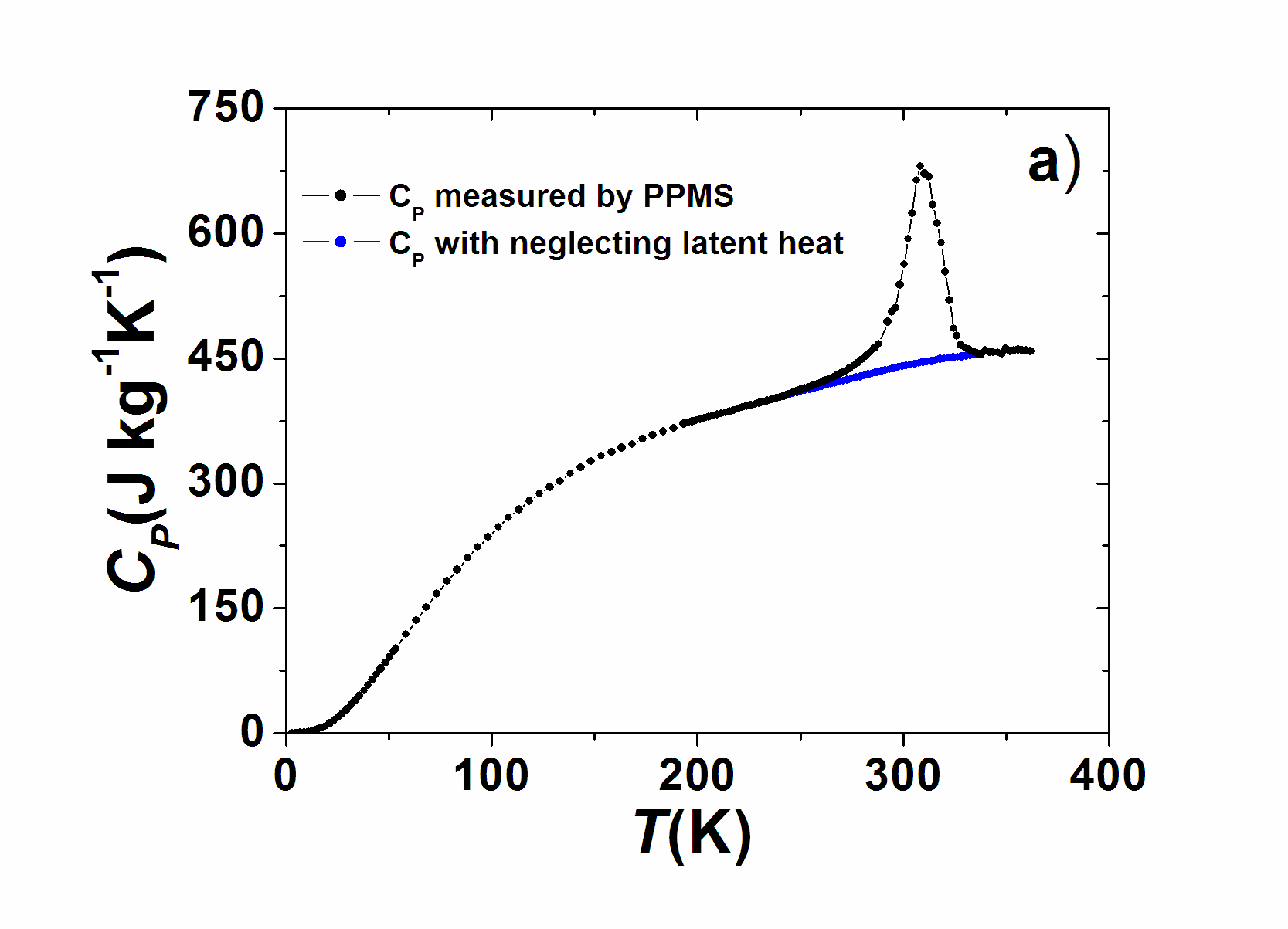


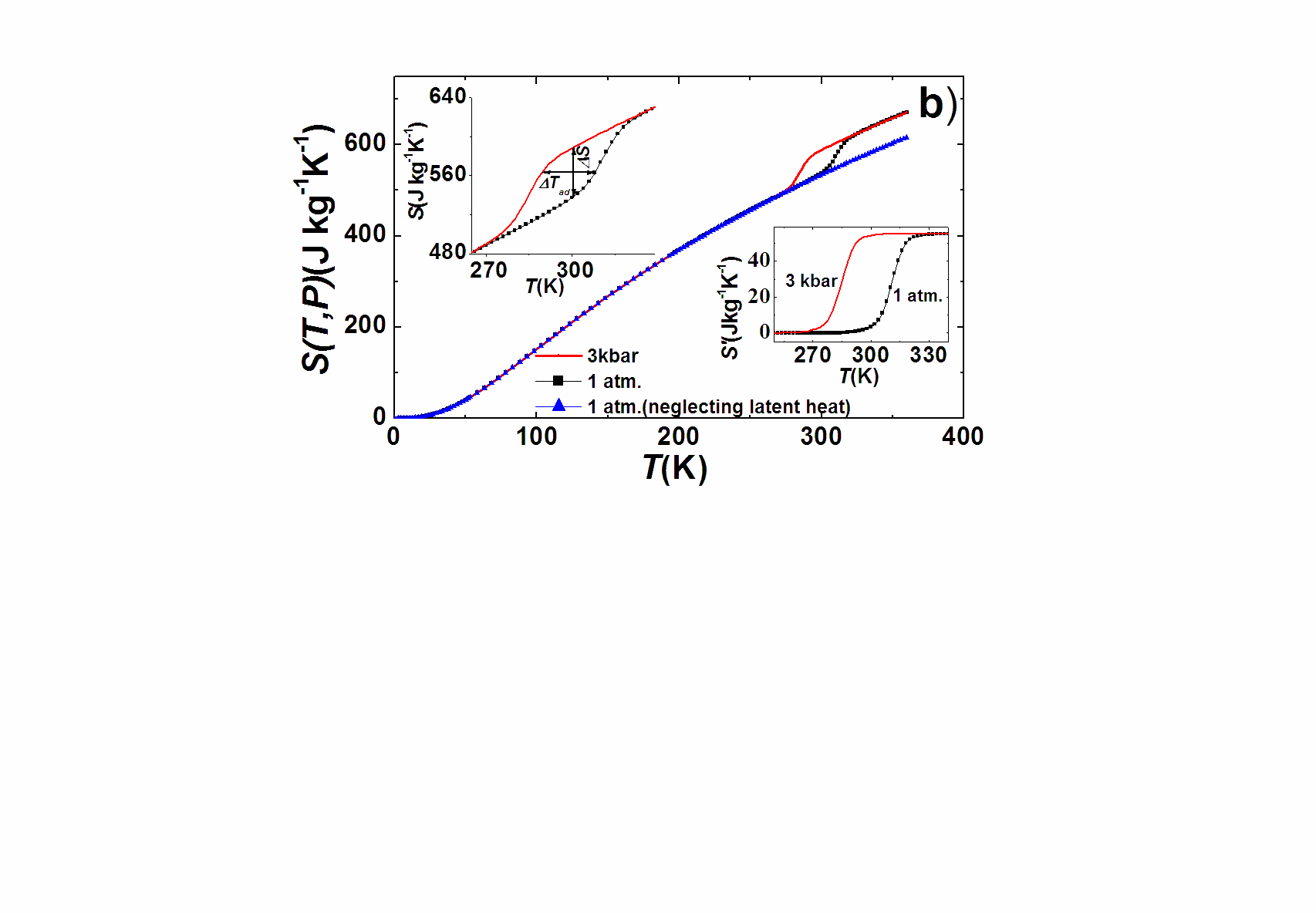


Fig. S3 a) Temperature dependence of the specific heat capacity (*Cp-T*) measured by PPMS from 2K to 360K under ambient pressure (black curve). The blue curve represents the *Cp-T*, where the *Cp* caused by the latent heat is neglected due to its inaccuracy in the phase transition region. b) Temperature dependence of the total entropy under different pressures (*S-T* curves). The blue plot is the basic *S-T* curve ignoring the contribution of the latent heat, while the black and red plots are the total entropy curves under ambient pressure and 3kbar, respectively. Upper inset shows the details of total entropy. Lower inset shows the entropy *S’-T* with neglecting the *Cp* contributions measured by DSC.

Direct DSC measurements under pressures have verified that a low pressure does not affect the *S-T* shape for the magnetocaloric materials with a similar magnetostructural transition such as Ni-Mn-In(the *S-T* curve with neglecting the basic *C_P_* under 2.6kbar is nearly parallel to that under 0.2kbar, see Fig.3a in ref.5), LaFeCoSi (the *S-T* curve with neglecting the basic *C_P_* under 2.1kbar is nearly parallel to that under 0 kbar, see Fig. 2 in ref.4), and Mn_3_GaN (the *S-T* curve under 0.93kbar is nearly parallel to that under 0 kbar, see Fig.2 in ref.6). From these *S-T* curves based on DSC measurements, entropy change *ΔS* can be reliably computed. [ref.4-6, *Adv. Mater.* 21, 3725 (2009)].

Previous experimental investigations on the MnCoGe-based compounds indicated that the *T_mstr_* exhibits a linear dependency on pressure (Anzai et al, Phys.Rev.B 18, 2173(1978); Niziol et al, J. Magn. Magn. Mater. 38, 205(1983))*.* Carton et al^[22]^ also found that an application of pressure to a similar composition Mn_0_*.*_93_Cr_0_*.*_07_CoGe can retain the transition slope and width within pressure *P*≤3.7kbar, and the *T_mstr_* almost linearly decreases with pressure. **Facts have proved that** the obtained *ΔS* using Clausius-Clapeyron equation for present system agrees well with that from the *S-T* curves (see following discussion).

**S5. Calculation of** *Δ****S* using Clausius-Clapeyron equation**

To verify the obtained entropy change *ΔS*, we also evaluated the *ΔS* by using Clausius-Clapeyron equation [Meyer et al, J. Phys. Radium 14, 82(1953); ref.35], the commonly accepted method for a system with the first-order phase transition. In such systems, the transition occurs if two phases have equal thermodynamic potential:

$[U_{1}-\frac{n_{1}M_{1}^{2}}{2}]-TS_{1}+(pV_{1}-HM_{1})=[U_{2}-\frac{n_{2}M_{2}^{2}}{2}]-TS_{2}+(pV_{2}-HM_{2})$ (1)

where *T* is the transition temperature under the pressure *p*, and *U_1,2_, S_1,2_, V_1,2_, M_1,2_* represents the internal energy, entropy, volume, and magnetization of phase 1 and 2, and *nM^2^* represents the molecular field contribution. Considering the negligible impact of a moderate pressure of 3kbar on either phase 1 or 2 [Fig.4, **line 5 // line 4, line 2 // line 1 in Fig.2a, and** the *ΔV*/*V* (~3.95%) of the two phases under 3kbar maintains nearly the same as the value(~3.9 %) under ambient pressure], it is reasonable to assume that a pressure of 3kbar only triggers the transition, but has little effect on the *S, M, V, n* values in either phase. Thus, the entropy change can be obtained as below,

$\Delta S=\frac{P\Delta V}{\Delta T}$ (2)

where *ΔV=V_2_-V_1_* is the change of unit cell volume across transition, and *ΔT* is the shift of transition temperature triggered by pressure. The evaluated *ΔS* using equation (2) is 56.6J/kgK under 3kbar based on the available *ΔV* and *ΔT* from Fig.S2a and Fig. 2a.

It has been reported that the evaluated *ΔS* error using Maxwell relation $\Delta S=\int_{H1}^{H2} \frac{\partial M}{\partial T}dH$ is in the range of 3-10% depending on the instruments if the accuracy of the magnetic moment *M*, temperature *T*, and magnetic field *H* was considered [Pecharsky et al, J. Magn. Magn. Mater. 200 (1999) 44]. Similarly, we evaluated the *ΔS* error for the both cases using Clausius-Clapeyron equation *ΔS=PΔV/ΔT* and based on DSC and *Cp* measurements (S4 above).

1. Using Clausius-Clapeyron equation, *ΔS=PΔV/ΔT,* the evaluated *ΔS* is about *ΔS=*56.6±2.8 J/kgK (error~4.9%) by taking the errors of pressure *P*~3% (neutron diffraction equipments in NIST, USA), *T*±1K(neutron diffraction equipments in NIST,USA), *V_H_*=77.031(3)Ǻ^3^,*V_O_*=160.161(12)Ǻ ^3^.
2. Based on the DSC and *Cp* measurements (S4 above), the evaluated *ΔS* is about *ΔS=*52.0±5.6 J/kgK (error~10.8%) by taking the errors of heat capacity *Cp*~0.5% (DSC-Q200 of TA instruments, PPMS-Quantum Design) and *T*~0.5% (DSC-Q200 of TA instruments, PPMS-Quantum Design). These errors were adopted through checking and comparing our measured data with the instrument manual.

**S6. Direct measurements of *ΔT_ad_***

Fig.S4 displays the direct adiabatic temperature change *ΔT_ad_* as a function of time measured by a Pt-1000 **thermometer** with a 3kbar pressure applied. The details can be found in the Methods section of manuscript.


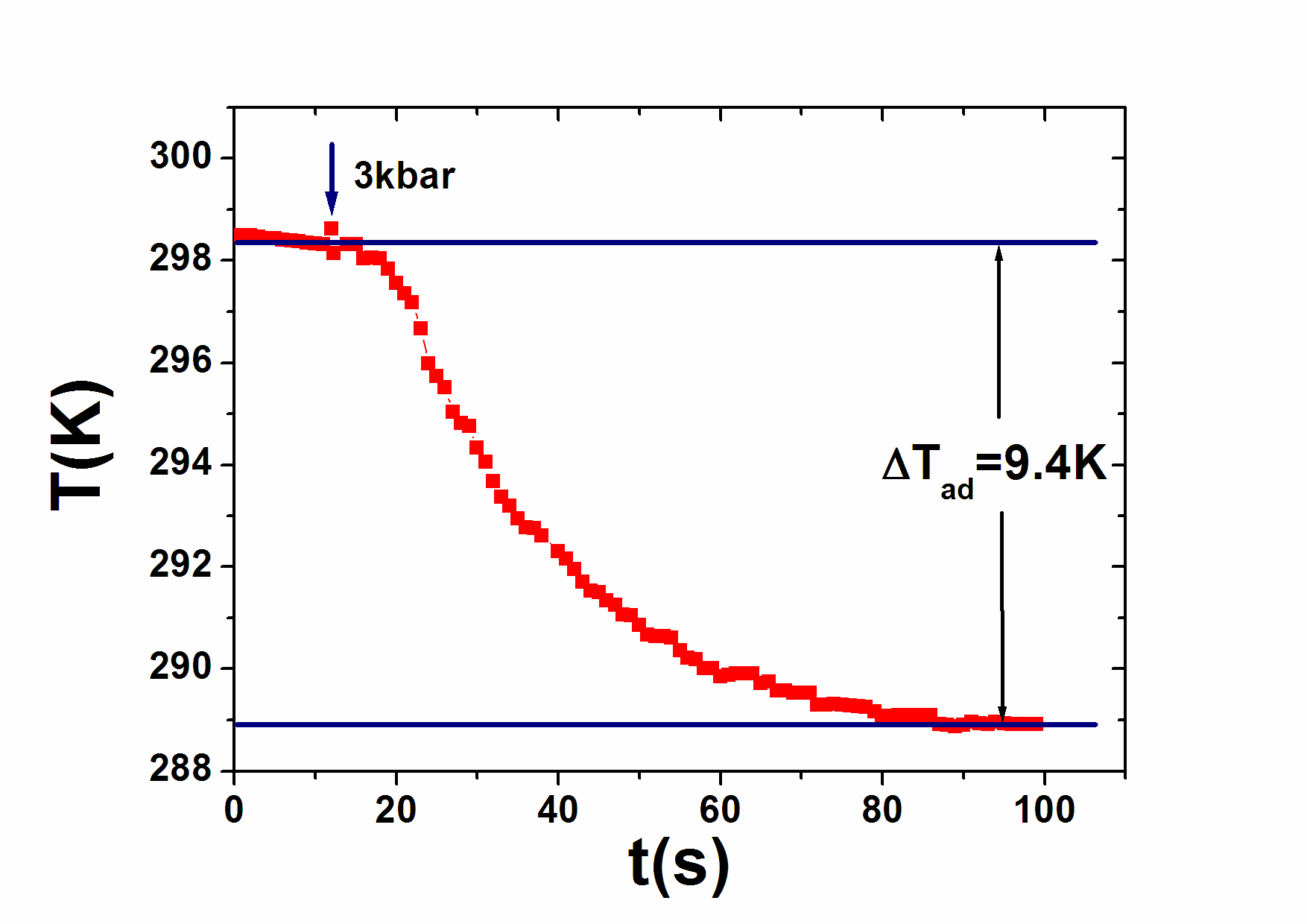


Fig.S4 The temperature measured as a function of time by a Pt-1000 **thermometer** under a 3kbar pressure.

**S7. Structure characters under pressure**

Fig.S5-1 presents the structure sketch from the view of XOZ plane for the Ni_2_In-type hexagonal structure of MnCoGe composition. The atomic distance of the nearest and the second nearest neighbors is denoted by *d1* and *d2*, respectively. In general, the Mn-Mn interlayer distance (*d1*) in the hexagonal structure is shorter than the Mn-Mn intralayer distance (*d2*) while the Co-Ge interlayer distance (*d2*) is longer than the Co-Ge intralayer distance (*d1*), as denoted in Fig.S5-1.


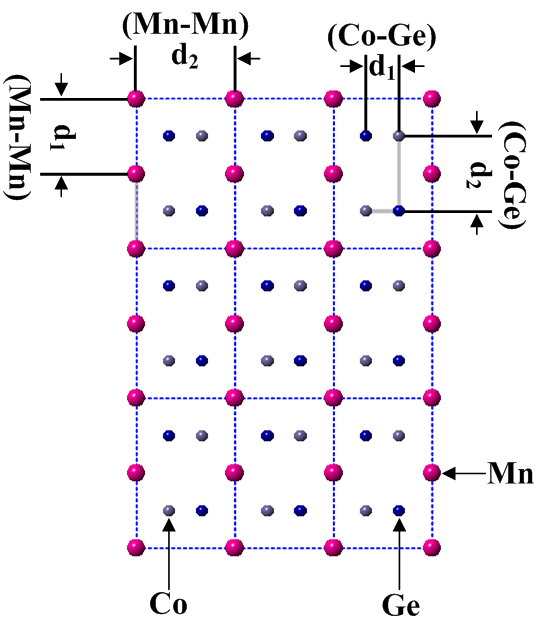


Fig.S5-1 The structure sketch from the view of XOZ plane for the MnCoGe hexagonal structure (*P6_3_/mmc*).


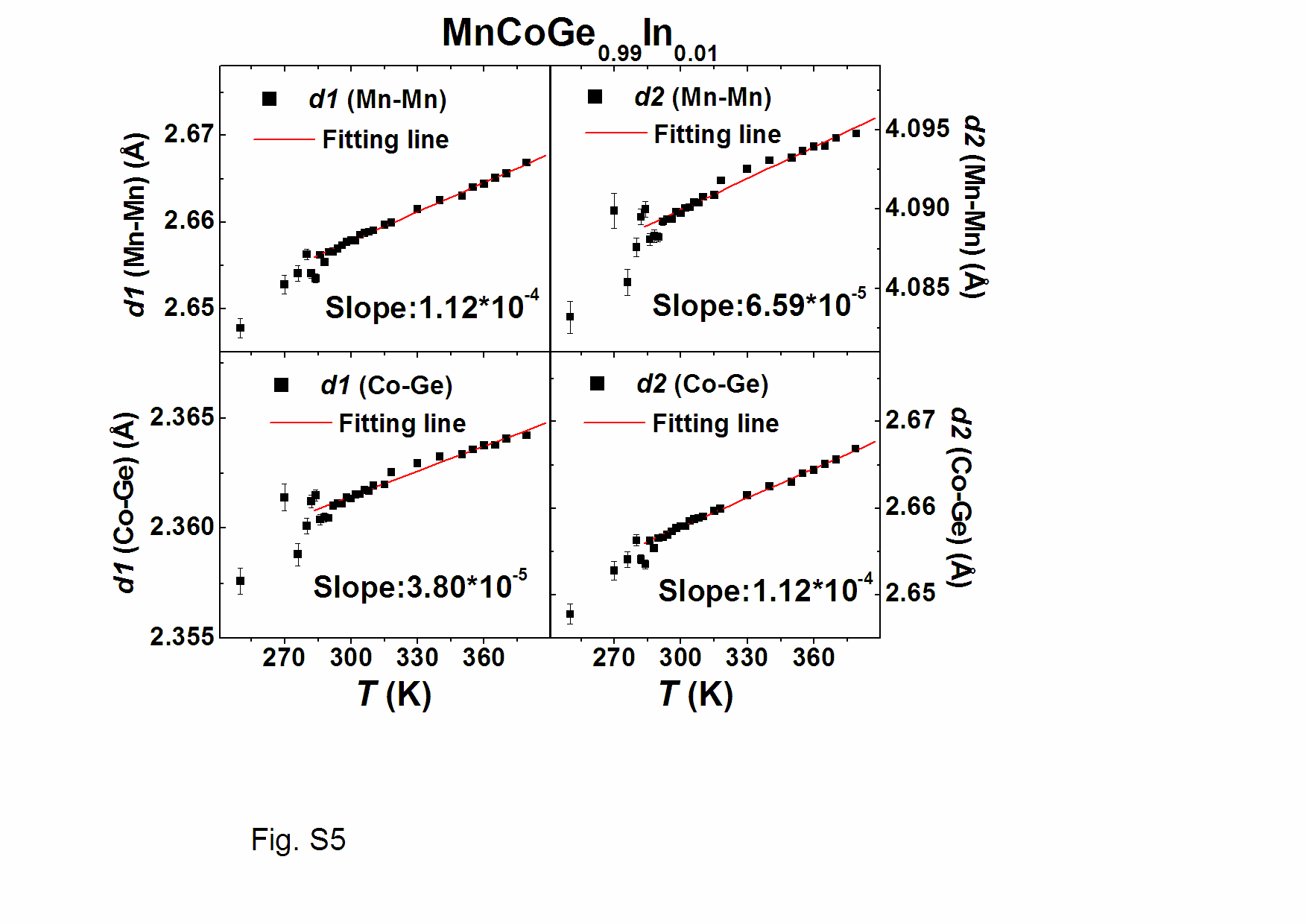


Fig.S5-2 Variation of **Mn-Mn/Co-Ge bond lengths with temperature**. The refined Mn-Mn/Co-Ge distances (with error bars), based on the high resolution neutron diffraction experiments, as a function of temperature measured under ambient pressure and zero magnetic field.


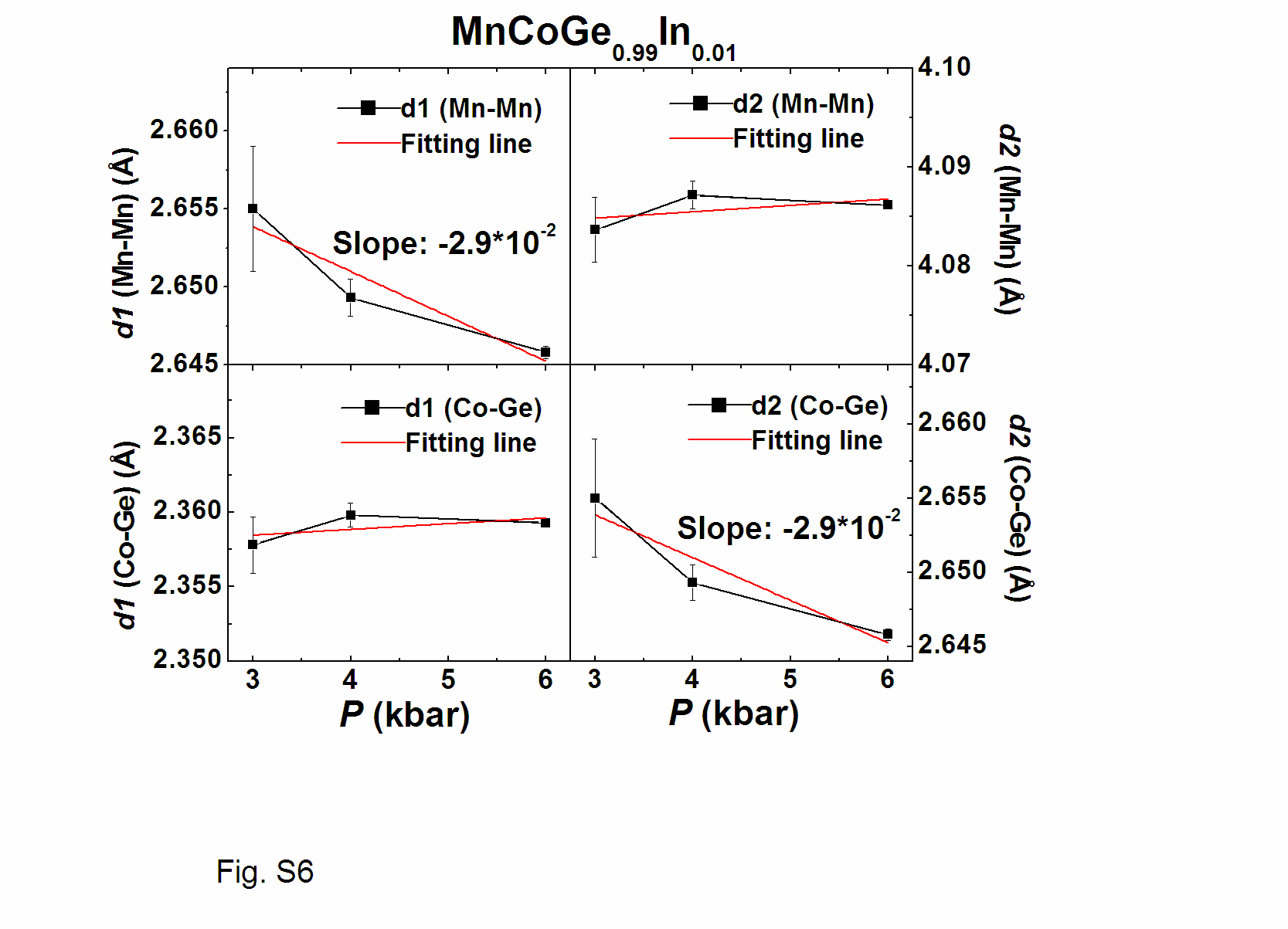


Fig.S5-3 **Mn-Mn/Co-Ge bond length with respect to pressure.** The refined Mn-Mn/Co-Ge distance (with error bars), based on high resolution neutron diffraction experiments, as a function of pressure measured at 259K.

Fig.S5-2 displays the refined Mn-Mn/Co-Ge distance with error bars, based on the high resolution neutron diffraction experiments, as a function of temperature measured under ambient pressure and zero magnetic field, while Fig.S5-3 shows the refined Mn-Mn/Co-Ge distance with error bars as a function of pressure measured at a constant temperature of 259K.
